# Putting the patient at the centre: a call for research involvement of nurses, midwives and allied health professionals working in genomics

**DOI:** 10.1136/bmjopen-2024-086962

**Published:** 2025-08-12

**Authors:** Lorraine Cowley, Sasha Henriques, J Roberts, Laura Monje-Garcia, J Nolan, K Lubasch, R Theobald, R Greer, N Fennell, A Clarkson, M Clapham, S Chilton, R Allon, Cheryl Stopford, Heather Hanson Pierce, D Holliday

**Affiliations:** 1 Northern Genetics Service, Newcastle Upon Tyne Hospitals NHS Foundation Trust, Newcastle Upon Tyne, UK; 2 Faculty of Education, Cambridge University, Cambridge, UK; 3 Engagement and Society, Wellcome Connecting Science, Hinxton, UK; 4 East Suffolk and North Essex NHS foundation Trust, Colchester, UK; 5 St Mark’s Centre for Familial Intestinal Cancer, London North West University Healthcare NHS Trust, Harrow, London, UK; 6 The Royal Marsden NHS Foundation Trust, The Institute of Cancer Research, London, UK; 7 Oxford University Hospitals NHS Foundation Trust, Oxford, UK; 8 Newcastle Upon Tyne Hospitals NHS Foundation Trust, Newcastle Upon Tyne, UK; 9 University of Cambridge School of Clinical Medicine, Cambridge, UK; 10 North Cumbria Integrated Care NHS Foundation Trust, Penrith, Cumbria, UK; 11 Leeds Teaching Hospitals NHS Trust, Leeds, UK; 12 Department of Paediatrics, University of Cambridge, Cambridge, UK

**Keywords:** Patients, Medicine, GENETICS, Health, Nursing research, QUALITATIVE RESEARCH

## Abstract

**Abstract:**

**Introduction:**

We report the collaborative views of a group of nurses, midwives and allied health professionals (NMAHPs) in the UK who have a genomics research remit or interest. Our group includes genetic counsellors under this diverse category of healthcare workers.

This group came together as part of the National Institute for Health and Social Care Research (NIHR) Genomics Research National Specialty Group. After responding to a survey to elicit the views of NMAHPs working in genomics, some of the original 45 respondents, along with others who learnt of the project by word of mouth, have worked together to produce this article.

**Objective:**

The paper aims to set out in clear terms the value of NMAHPs to research that supports the patient-centred implementation of genomics in the National Health Service (NHS).

**Key argument:**

We discuss four potential areas where NMAHPs, in particular, can contribute to the research. These are patient perspectives and epistemic justice, psychosocial impacts, the familial nature of genomics and equity. We argue that this group (NMAHPs) represents a potentially underused resource for the NHS as it seeks to ensure that advances in genomics are translated into patient benefit.

**Conclusions:**

We propose that NMAHPs, with our research expertise, are well placed to shape and deliver a research agenda that explores models of patient-centred care in the genomics era. We call for increased funding for NMAHP research roles and funding opportunities to deliver this fundamental work.

The patient will be at the heart of everything the NHS [National Health Service] does.^1^

## Introduction

To truly place patients at the centre of genomic medicine, research exploring the wider application of genomics in mainstream medicine is essential to improve services. As nurses, midwives and allied health professionals (NMAHPs) in genomics, we are primed to deliver this research through the co-production of knowledge with patients, accounting for their needs, preferences and values.

## Who we are and how we came together

In 2021, a survey was conducted to elicit the views of NMAHPs specifically working across a range of disciplines in UK healthcare, directly and indirectly implementing genomic medicine. The survey was conducted by NMAHP representatives on the National Institute for Health and Social Care Research (NIHR) genomics specialty group.

Since March 2022, we have had six online meetings with representation from the original 45 survey respondents, plus others who had learnt of our network by word of mouth. In 2023, 12 of us came together for a writing workshop where we refined and clarified our views that are set out in this paper.

## Genetics in the past

Historically, patients accessed genetic testing in the NHS through specialist regional genetics services and were supported through their genetic testing journey by consultant geneticists (medical doctors with specialty training in genetics) and genetic counsellors (nurses, midwives or other allied health professionals with specific scientific and counselling training). This early delivery model, with our understanding of genetic aetiology in its infancy, was driven by phenotypic presentation, primarily aiming to make a molecular diagnosis of monogenic conditions in symptomatic individuals, before cascading testing to at-risk relatives. Genetic testing was limited to single genes or targeted small panels. The laboratory work, data analysis and variant interpretation were expensive and time-consuming. Combined, these limitations meant that many families undergoing genetic testing did not receive a molecular diagnosis.

## Present and future genetics

The provision of genetic testing in the NHS is undergoing a period of significant change.^2^ Some of this is driven by the rapid increase in speed and reduced costs of sequencing. The availability of whole exome and genome sequencing has led to clinical genetic testing moving away from single genes, instead offering testing via panels that test multiple genes at once. This development has increased the molecular diagnostic yield for inherited conditions.^3^,^5^ It has also meant that the complexity of the tests available has increased.

In addition, the widest ever breadth of non-genetic clinicians across the NHS can now directly offer genomic tests to patients at the point of care. This is often referred to as ‘mainstreaming’.^6^ Test criteria, including which specialists are authorised to order the test, are set out in the National Test Directory.^7^

Genomic testing is also used increasingly for the prediction of risk. Historically, this has been limited to a reasonably small number of highly penetrant Mendelian genes. Research is ongoing to understand the clinical utility of a wider range of genetic tests, including polygenic risk scores, which have the potential to aid personalised risk stratification when used with other data, such as lifestyle information, family history and results of monogenic tests.^8^

The available avenues through which individuals can receive genetic information have rapidly increased,^9^ including genotype-first approaches, such as additional findings from the 100 000 Genomes Project, newborn screening for genetic conditions (the Generation Study) and the abundant commercial availability of ‘direct-to-consumer’ genetic tests. The variety and purpose of genetic testing are also increasing beyond diagnosis. For example, pharmacogenomics is driving personalised treatment and improving drug safety and efficacy.^10^

## Making space for NMAHPs to do research

The NHS continues to be at the forefront of clinical research, capitalising on the UK’s position as a global leader in genomics research. The importance of developing research capacity and culture has become an important priority in the NHS. This has led to calls for a focus on encouraging participation in research by professionals other than doctors, as they represent most of the healthcare workforce.^11^

NMAHPs encompass a wide range of healthcare professionals (HCP) who are integral to patient care. Nurses and midwives are often the first point of contact within the healthcare system. Allied health professionals are a diverse workforce, including physiotherapists, occupational therapists, radiographers and more. For the purpose of this review, we included genetic counsellors under this umbrella, while recognising that in some NHS bureaucracies genetic counsellors are officially categorised under healthcare sciences.

The generalised call for more research within the NHS is reflected in the published research strategies for Nurses,^12^ Midwives^12^ and Allied Health Professionals.^13^ All these strategies clearly identify ambitions to transform the culture around research and evidence-based practice.

There is widespread recognition that increasing research activity around NMAHPs can support high-quality research as well as facilitate the implementation of research findings into practice.^14^ However, opportunities for NMAHPs to contribute to research are limited compared with their medical colleagues. NMAHPs’ progress compared with that of medics has been slow, with fewer clinical academic career opportunities where research is routinely expected and undertaken as part of practice.^15^

The 2016 strategy document from the NIHR states:

While integrated clinical and research career pathways exist for medical professionals, comparable opportunities are not well defined for NMAHPs, yet they form the vast majority of the clinical workforce which provides care to patients.^16^

Cooke *et al*’s^16^ mapping of research capacity activities supporting non-medical professionals found that NMAHP managers have little experience in supporting clinical academic pathways. It further found that NHS career structures for clinical academic posts are inconsistent at best and more often non-existent.

We believe that maximising the patient benefit of new genetic tests requires placing patients at the heart of implementation and policy. Furthermore, we argue that NMAHPs in genomics represent a powerful resource that exists within the NHS to drive research that ensures that technological innovation leads to real and equitable patient benefits.

## Implementing genomics

The future of genomics is often portrayed as sunlit uplands and boundless opportunity. However, several challenges and barriers have been identified to its successful implementation. Some of these are practical and procedural, such as the administrative aspects required to carry out the consenting process and ordering of genomic tests. These issues are often exacerbated by the lack of a trained and available workforce within the NHS to take consent, arrange sample logistics, interpret findings and communicate the results of genomic tests. Other barriers identified include the challenge of accurate phenotyping and the lack of a centralised system in the NHS that makes collating trio samples (child and both parents) and forms challenging.^17^

NMAHPs have and will no doubt continue to contribute to important research in these areas. Here, we outline four specific research challenges and opportunities that exist in the implementation of genomic medicine into mainstream care. Alongside an explanation of these issues, we explore how NMAHPs can contribute to research in these areas.

The four areas of focus are patient perspectives and epistemic justice, the psychosocial impacts of genetic tests, the familial nature of genomic testing and equity.

We focus on these four issues not because we believe they hold any greater status than others do but rather because they represent exemplars; they provide prescient and compelling examples of where there are opportunities for NMAHPs to contribute to solutions through research. We will consider these in more detail in the next section.

## Patient perspectives and epistemic justice

Putting the patient first means ensuring patients’ voices are represented. It is essential that we use meaningful Public and Patient Involvement and Engagement (PPIE) in shaping the direction of research from the outset. While PPIE groups provide an invaluable perspective and contribution to this, voices that do not engage with these groups may be missed. The work of NMAHPs in genomics complements the work of PPIE groups in research, in that our research can access voices who may not otherwise engage in PPIE.^18^ Understanding what information and support is helpful to patients and how patients would like that to be delivered will be vital in determining how we develop models of genetic testing across the NHS.

Research that explores patient perspectives is also important in the genomic space to address epistemic injustice. The concept of epistemic injustice was introduced by Fricker and defines how people can be devalued as *knowers*.^19^ Fricker identifies two key forms of the concept. The first is *testimonial injustice*, which refers to cases where testimony is unduly dismissed because of prejudiced beliefs regarding minority groups. The second is *hermeneutical injustice,* referring to cases where a community’s shared vocabularies have been structured in a way that unfairly distorts or stifles understanding for, and of, a minority group.

Within clinical genetics, the concept of epistemic injustice is relevant in exploring how differences and disabilities are defined and treated. People from the deaf community, the autistic community and those writing from a disability rights perspective, for example, have all argued that genetic testing can serve to reinforce discriminatory attitudes and prejudicial medical models of disability.^20^,^22^ For the implementation of genetic testing to be fair and equitable, it is important that a plurality of voices be heard in policymaking. This can ensure that we avoid epistemic injustice as we draw boundaries between disability and difference—the normal and pathological—and how these boundaries relate to the acceptable use of genomic testing in the NHS.

Tackling epistemic injustice requires recognising the plurality of viewpoints and gathering patient experiences in a way that generates high-quality, methodologically rigorous evidence.^23^ The professional identities of NMAHPs, in particular, lend themselves to research in this area. Genetic counselling and nursing, for example, have professional identities that are patient-centred. Within genetic counselling, this is reflected in the counselling and communication skills developed by practitioners. Genetic counselling practice often draws on the epistemological belief that the meaning of a genetic test is not transferred from counsellor to patient but is co-created with them. More generally, tackling epistemic injustice will require working with and involving a wide range of communities. The NMAHP workforce spans across the healthcare ecosystem—from community to hospital—and thus represents a group well-placed to conduct research that invites patients to voice their authentic experiences, providing opportunities for their lived experience to influence service improvements. Within the NMAHP community, there is an untapped resource of professionals with suitable skills ready to tackle issues of epistemic injustice.

## Psychosocial impacts

Genetic testing can deeply affect some individuals psychologically and socially. Often, genetic tests require careful consideration, with an exploration of the patient’s needs, beliefs, values and expectations. This is particularly the case if testing is being considered in pregnancy.

The psychological implications of a test mean that appropriate support during genetic testing may need to include help with decision-making, talking to family members, coping with previous familial experiences of a condition and recognising signs of complex grief that need additional input from other specialised psychology services.^24^,^27^

For many diagnostic and predictive genetic tests, HCPs will need to attend to these psychosocial components, providing psychological care and supporting adjustment following any genetic diagnosis.^28 29^ Patients may also need support to understand the nuances of not finding a genetic cause or diagnosis. This can be difficult, as the expectations surrounding genetics often stem from ideas about the power and certainty of DNA and genetic information.^30^ As genetic testing becomes more complex, the psychological implications of increased uncertainty are not yet understood. Mainstreaming of genomic testing can introduce a new type of ‘odyssey’ for patients and/or relatives, such as when there are uncertain findings.^31^

Patient-centred care, as it relates to psychosocial needs, also means ensuring that patients are offered the appropriate depth or intensity of discussion either before or after a genetic test. The terms ‘genetic test’ or ‘genomic test’ are umbrella terms that refer to a diverse range of tests carried out for a wide range of purposes. While prenatal testing, for example, will always require genetic counselling, there are a good number of genomic tests that are unlikely to require pretest or post-test genetic counselling. For example, a point-of-care pharmacogenomics test may be carried out to determine chemotherapy choice. This test may have a few implications beyond the type of chemotherapy offered. Genetic counselling—at least in its traditional conception—is unlikely to be needed or appropriate in this case. To ensure high-quality patient care and effective resource management, it is important to understand what types of tests can be implemented with a lighter touch, at least in reference to the counselling required. The question of *who needs genetic counselling* is given more urgency with the increasing number of ‘genotype first’ tests, such as newborn screening, where genetic tests are done at the population level on unaffected individuals. In these cases, patients may receive unexpected genomic information.

The integration of genetic testing into the NHS will require a nuanced understanding not just of its purpose but also of the psychosocial impacts. We do not know what these psychosocial impacts will be, how best to manage them or which HCPs are best placed to support patients in a healthcare system where integrated mainstream genomic testing is common. NMAHPs working in genomics are embedded within practice and have the benefit of day-to-day experience with the changing face of genomics in healthcare. This breadth of clinical experience means that NMAHPs are ideally suited to contribute to research that explores the psychosocial impact of genomics tests.

## Familial nature of genomics

An additional aspect to attend to, as genomics is integrated into other medical specialities, is the familial implications of a genetic diagnosis.^32^,^34^ The familial nature of genetic knowledge is demonstrated in the first statement on the record of discussion for genomic testing in the NHS. This states:

The results of my test may have implications for me and members of my family. I understand that my results may also be used to help the healthcare of members of my family and others nationally and internationally. This could be done in discussion with me or through a process that will not personally identify me.^35^

The heterogeneity of genetic tests—in both purpose and type—along with the complexity of family dynamics, means that considerable ethical and policy issues arise. As genomics has become a routine part of mainstream care, the familial nature of genomics continues to receive sustained attention.^36^,^38^ Here we outline some of these areas to demonstrate the potential value that NMAHP’s increased participation in research could address.

For some patients, the familial nature of a test may generate a significant amount of extra anxiety about the impact of results on family dynamics and relationships.^39^ Additional personal and familial pressures may be experienced by the first person to receive a genetic diagnosis, since they often have the task of sharing the information with the rest of the family,^40^ while navigating their own new diagnosis.^41^ Cascading genetic testing to at-risk relatives allows family members to make active choices towards their healthcare, often before they even develop symptoms. While there are challenges to sharing information within families, if clinicians do not facilitate cascade testing, this is a missed opportunity to potentially prevent disease.^42^

However, this focus on sharing information may be less familiar to those outside of clinical genetics. In recent years, the definition of ‘autonomy’ in genetics practice has moved towards a relational rather than an individual concept.^43^,^45^ This may feel less familiar to those used to a more individualised concept of autonomy. As such, HCPs, more widely, may feel less confident in knowing how to support patients in sharing information. Commonly, genetic counsellors and nurses support patients as they share information with their wider family. Given this clinical experience and expertise, NMAHPs are well-placed to generate research that can support both patients and staff as they seek to deal appropriately with the familial implications of genomic tests.

## Equity: how do we ensure that testing is available to all?

Ensuring equitable access to genomic healthcare is paramount to the NHS’s genomics strategy. Genomic studies face well-described challenges around dimensions of diversity, which complicate and limit prediction and utility, impacting our clinical practice.^46^,^48^ Race, gender, socioeconomic group and disability all affect how patients access and experience genomic services.^49 50^ Within research, some groups have historically been, and remain, significantly under-represented, which, in turn, often affects clinical care.^51^ The burden of rare and genetic diseases then becomes greater on these under-represented and underserved groups leading to increased healthcare costs and reduced quality of life.^52^

Equitable delivery of genomic medicine has been identified as a priority in a number of UK-wide projects and strategies. For example, the first-ever NHS genomics strategy, ‘Accelerating Genomic Medicine in the NHS,’ set out ‘delivering equitable genomic testing for improved prediction, prevention, diagnosis and precision medicine’ as one of its four main priorities.^53^

The value placed on equity is also present in some significant research projects, such as the Generation Study. This is a project run by Genomics England and delivered in the NHS. It is offering whole genome sequencing to 100 000 babies at birth. It will assess the potential utility of whole genome sequencing to screen for treatable, early-onset genetic conditions. For a condition to be included in the study, it must meet four criteria. The last of these states:

Conditions screened for are only those for which the interventions are equitably accessible for all.^54^

The barriers to equitable care are varied and complex. They include institutional and structural barriers as well as attitudinal factors. For example, some groups in society are less likely to see the value of genetic testing or trust in institutions delivering the care.^55 56^ NMAHPs represent a highly diverse workforce. As such, they are a potentially powerful tool at the NHS’s disposal for researching barriers to equitable care in genomics, as they cut across the multiple dimensions of healthcare. NMAHPs’ diversity—both in terms of who they are and their distribution across the landscape of the NHS—makes them a powerful group to leverage in understanding inequality through research.

## Why is an NMAHP research network important?

We have outlined four areas where we believe NMAHPs could provide valuable input into research. In the final section, we will outline some barriers to research and what our group can offer.

There is a growing appreciation for the value of NMAHPs in research. In their 2021 review, Jones and Keenan called them ‘a new powerhouse within clinical research’.^57^ At the same time, it is estimated that only 0.1% of the NMAHP workforce are currently in clinical academic roles.^58^

One approach to improving NMAHPs’ contribution to research is through the appointment of clinical academic roles. Many of these have been supported by the NIHR. These roles balance time between clinical work and research. While clinical academic roles are valuable, on their own, they are not likely to shift the scale of NMAHPs engaging in research significantly. Other approaches will be required, especially those that focus on allowing NMAHPs to contribute in a wide variety of ways.

Whitehouse *et al* propose a framework based on building the capacity, capability and confidence of NMAHPs in clinical practice to contribute to research. They argue that this will breed a culture where NMAHPs are confident to develop and enact their own innovative ideas, with a higher percentage becoming research-active.^59^

We have reported the views here of a nascent research network of NMAHPs who have identified a common goal and purpose, the desire to improve and champion NMAHP research within the genomics space. We have set out four possible areas where we feel NMAHPs could contribute positively to research. However, to achieve these goals, we argue that changes are needed.

Some of these changes are attitudinal and cultural. NMAHPs have often suffered from confusion about where they ‘sit’ within higher education institutions and the NHS. Whereas ‘clinical academic’ is a well-established role, this is not so for many NMAHPs. This is reflected in NHS culture, where there can be a reluctance to ‘release’ people from NHS duties.^57^ Too often, NMAHPs encounter embedded beliefs that research and clinical duties are seen as oppositional rather than symbiotic.

We urge policymakers, funders and NHS managers to consider how they can support NMAHPs in contributing to research in genomic medicine, particularly in the four key areas we have discussed. To enable NMAHPs in genomics to produce high-quality research, dedicated time and financial resources are imperative.

Our group is well placed to champion the implementation of research findings in clinical practice. We aim to expand our network to facilitate equitable, evidence-based outcomes for patients across the entire NHS.
