# Supplementary material for: Putting the patient at the centre: a call for research involvement of nurses, midwives and allied health professionals working in genomics
Source: BMJ Open. 2025 Aug 12;15(8):e086962. doi: 10.1136/bmjopen-2024-086962 (PMC12352142; doi:10.1136/bmjopen-2024-086962)
Supplement: Supplementary file 1 [file bmjopen-2024-086962.reviewer_comments.pdf]

PEER REVIEW HISTORY

BMJ Open publishes all reviews undertaken for accepted manuscripts. Reviewers are asked to complete a checklist review form and are provided with free text boxes to elaborate on their assessment. These free text comments are reproduced below.

ARTICLE DETAILS

Title (Provisional)

Putting the Patient at the Centre: A Call for Research Involvement of Nurses Midwives and Allied Health Professionals Working in Genomics

Authors

Cowley, Lorraine; Henriques, Sasha Louise May; Roberts, J; Monje-Garcia, Laura; Nolan, J; Lubasch, K; Theobald, R; Greer, R; Fennell, N; Clarkson, A; Clapham, M; Chilton, S; Allon, R; Stopford, Cheryl; Pierce, Heather Hanson; Holliday, D

VERSION 1 - REVIEW

**Reviewer**	**1**
**Name**	**White, Stephanie**
**Affiliation**	**University of Technology Sydney**
**Date**	**13-Aug-2024**
**COI**	**Nothing to declare**

Putting the Patient at the Centre: A Call for Research Involvement of Nurses Midwives and Allied Health Professionals Working in Genomics

Overall comments

Thank you for the opportunity to review this manuscript that highlights the importance of NMAHPs conducting genomics research to deliver evidence-based, patient-centred care. The authors set out a number of reasons why NMAHPs should be involved in research and the challenges faced in delivering person-centred care.

The article would be improved with a more transparent description of their survey, how this is informing the article, and whether they have/need ethics approval to report this data. They also refer to 'discussions' informing the article, and these could be described in better detail (e.g., how many meetings were held, when did they occur, who attended etc).

One aspect I felt was missing from the article was the interplay between clinicians, clinician-researchers, and academic researchers, and what opportunities and challenges exist within these structures. The authors mention time as a barrier to

conducting research, but there was no explicit discussion about how networking with academic institutions could facilitate research opportunities for those who are primarily working in the clinical setting. Further to this, it's not clear whether the authors are coming to this discussion from an academic or clinical perspective, and this would be helpful to understand in order to contextualise their article.

The other missing component was where NMAHP research fits in within the larger body of genomics research. The arguments for increased participation would be strengthened by highlighting what it is specifically that we add to the research agenda. You discuss addressing the psychological and familial aspects of genetics, but it would be good to articulate what our unique lens/training/viewpoint adds to the research already undertaken/underway by other disciplines. You could also highlight the limitations of approaching research through our lens and how we can overcome the challenges (e.g., limited opportunities to lead research groups, or inadequate research training in undergrad/post grad education).

Overall, I believe this is an important topic and article, but it requires some refinement before publishing. I hope these comments are helpful and I would be happy to review another version in the future.

Abstract

Line 49: Is it accurate to say this an unprecedented approach? I can think of many articles that report on group discussions/consensus.

Figure 1.

The figure or legend could use a little more explanation, perhaps by including the question that was asked alongside the figure. I am assuming that when you say the responders are "published", this means they have conducted a research study and published this in a peer-reviewed journal? A clearer explanation would help. The same goes for 'opted into email group' (not clear what email group you mean or why this is important data to showcase up front) and 'in education or training' (does this refer to entry level qualifications for their profession?).

You also mention that other people came to this discussion who did not respond to the survey, so is there further information about who the group ended up being that contributed to this article?

Note for self: is this data that they have published previously or should they make some reference to whether ethics approval was obtained for this collection and dissemination of data?

Page 7, line 39

You make reference to your survey here and elsewhere, but it's not clear to me whether this article to seeking to report the findings of your survey because you have not provided concrete data, or referenced a previously published study in which your data is described. I think you need to make your intentions explicit somewhere within the article because it's also not clear whether you are meeting ethical requirements around reporting this data. Do you have ethical approval to report data from this survey, or did you receive an exemption from a relevant ethics review committee?

Page 7, lines 49 – 60

It's great that the benefits of qual research are highlighted here, but it would be good to complement this with the strengths of quant research and perhaps mixed-methods, particularly as MM are recommended by groups like the Medical Research Council as the preferred methodology for complex interventions and implementation research.

Page 8, first two paragraphs

I agree that these are important considerations. In the context of this article, which seeks to advance the involvement of NMAHPs in genomics research, this section would be improved by linking how these challenges relate to the involvement of NMAHPs in research.

Page 10, line 7-9

Can you describe further how NMAHPs are able to reach underrepresented voices that PPIE groups are unable to?

Pag 10, lines 28-30

This section would be strengthened with a more specific description of what your group intends to do. For example, how will you champion the implementation of research findings into practice? Who will you expand your network to and how will this facilitate the outcomes you want to achieve?

**Reviewer**	**2**
**Name**	**Siermann, Maria**
**Affiliation**	**KU Leuven**
**Date**	**28-Aug-2024**
**COI**	**N/A**

General:

- This is an interesting commentary paper that highlights the need for patient-centred research in genomics and for the involvement of NMAPHs in that research. I think the

patient-centred research (and care) is very important, particularly in the mainstreaming of genomics, and therefore commend the efforts of the authors.

- However, the structure of the manuscript could be improved. The aim of the paper is not fully clear. Sometimes it discusses the need for more patient-centred care, sometimes for more patient-centred research, sometimes for more research to be done by NMAPHs, and sometimes for more research to be done by the group of authors. Of course these issues are interrelated, but the specificities and interrelations of these issues are not made very clear. It might be better to choose one area to focus on specifically, and to really argue for that issue specifically (and make the interrelations with the other issues clearer).

- The aim and content of the survey is not very clear to me. As I have stated in a comment later on, I would opt to either focus more on the results of this paper, or otherwise to leave the survey out and write the manuscript as a commentary paper. This might help streamline the paper and emphasize its goal.

Abstract & title:

- In the abstract you conclude that "We propose that NMAHPs, with our research expertise, are best placed to shape and deliver a research agenda that explores models of patient centred care in the genomics era. We call for increased funding of NMAHP research roles and funding opportunities to deliver this fundamental work." However, this is different from your conclusion in the paper, where you talk more about ways the move forward, and do not state that NMAHPs are the best placed, but rather that it is crucial that NMAHPs are involved in research and clinical care in genomics.

Introduction:

- Maybe I'm missing something, but I don't see the footnote information for the quote "The Patient will be at the heart of everything the NHS does"1. Who is this quote by?

- The introduction is a bit vague and short. I would suggest starting with describing a development or 'problem', to make the aim and need of this paper clear.

- Or integrate it more with the sections that follow, instead of having separate section/subheadings.

Present and Future Genetics:

- "Evidence about psychosocial impacts of these new types of tests and their consequences for individuals in families who decline testing is limited9.": What do you mean with 'these new types of tests'? For some of the newer tests you mention earlier there has been research done about the psychosocial impacts of these tests. Can you discuss some of these impacts (with references)?

- "A recent systematic review of direct-to-consumer genetic tests found consumer satisfaction with these products, healthcare professional scepticism, false positive results, and occasional harm10.": This sentence is difficult to follow. Can you say a bit more about

the type of direct-to-consumer genetic tests you are referring to? And say more about what you mean with scepticism, satisfaction and harm?

Why is an NMAHP research network important?:

- "Numerous projects around patient experience, evaluation and service development were identified by responders to our NMAHP survey": What do you mean by this? Do you mean projects of the authors of this manuscript? Or are you talking about projects in general? Can you give references?

- "In addition, our group has research expertise in variant interpretation12, clinical outcomes13, 14, guideline development14, 15 and data management16." and "We expressly assert our readiness to lead on patient centred genomics research, namely, combining quantitative with qualitative approaches. Excellent qualitative research gives patients opportunities to voice authentic experiences. It provides rich datasets and real-world patient insights upon which to base service improvements. We are acutely aware of the skills needed by qualitative researchers and the need to demonstrate reflexivity. Our training and practice develop these skills, which are essential for approaches such as grounded theory, interpretative phenomenological analysis, interview skills and ethnography." The paper seems to jump from emphasizing the importance of patient-centred research and the role NMAHPs could have in this research in general, to arguing for you as a group specifically being qualified for that research. I think the paper might be stronger by focussing less on your skills as a group, but more on the need for patient-centred research in general and what NMAHPs could add to that research.

Challenges to Patient Centred Care - Putting the Patient First:

- "Cascading genetic testing to at-risk relatives allows family members to make active choices towards their healthcare, often before they even develop symptoms. If cascade testing is not facilitated by clinicians, this is a missed opportunity to potentially prevent disease31.": I think it would be important to also highlight the fact that not every family member would want to know and be tested. While it can be said that having the option to get tested is important, I think the option of choice is important too. In that sense, getting tested should not only be about disease risk, but also about informed choices. I am also not sure what you mean with the potential to 'prevent disease' in genetics? Are you referring to reproductive selective options?

- Other than that, this section reads really well and seems to be the key part of this manuscript.

Equity: How do we ensure that testing is available to all?:

- The transition from the previous section to this section could be improved.

- It would be interesting to highlight more that not just access to genetics sercives is not equally available, but that genetics research itself does not ensure equity (think for example

about the limited representation of people that are not of European ancestry in biobanks used for genome-wide association studies).

Making space for NMAHPs to do research:

- It is not clear to me what exactly was asked in the survey and how this “demonstrates a skilled and motivated workforce who are well placed and equipped to produce research that can meet the challenges set out above”. I am not doubting that the NMAHPs included are indeed this skilled and motivated workforce, but I am confused about how the survey shows this. Perhaps this survey is not relevant enough for the point you want to make. I think you can argue that putting the patient at the centre of genomics research at the NHS and the need to value the skills of NMAHPs to do just that would be enough of an argument, without a need for the survey.

- Or the other option was to spend more time on the survey results. Now you something refer to these results, but as a reader the results (and aim) of the survey is not very clear.

- Page 10, line 10: Do you mean ‘PPIE’ rather than ‘PPI’?

Conclusion:

- I like that you discuss potential ways to move forward in your discussion.

- However, improving clinical care and patient-centred research seem to be discussed interchangeably. While I realize that research and care goes hand in hand, I think it is important to be clear in what you are discussing and what issue(s) you want to raise.

- While not discussing research in genetics specifically, perhaps the paper ‘Ethical preparedness

in Genomic medicine: how NHS clinical scientists navigate ethical issues’ by Sahan et al. 2024 and our commentary response ‘Stimulating professional collective responsibility from the outset in

mainstreaming genomics’ could be useful for your manuscript, since similar issues are discussed, though with a different emphasis.

**Reviewer**	**3**
**Name**	**Puddester, Rebecca**
**Affiliation**	**Memorial University of Newfoundland**
**Date**	**13-Sep-2024**
**COI**	**I have no competing interests.**

Thank you to the team for the opportunity to review this commentary article. I think the crux of the arguments of the article are well-worth making.

As a nurse who uses methods of patient engagement in genomics healthcare research, I agree that NMAHP can add tremendous value in the space of engaged/co-design research by virtue of their clinical training and skills that can contribute to person-centred cultures of care. I also believe that there is a need for a culture of effective and two-way transfer of knowledge between research and practice which requires protected time and infrastructure for both activities.

I do think there are a number of things that can be clarified/streamlined to improve this paper and its argumentation. At times, there was repetitive rhetoric that could be enhanced with supportive evidence. Thus, I offer my suggestions in the spirit of collegiality.

Page 4 Line 30- It is not clear to me the details surrounding the survey, why was it sent out, to who sent it out? Was there an option to leave contact information resulting in the formation of this group? I think that more detail surrounding the motivations and circumstances surrounding how this authorship group came together would make it a lot clearer for the reader.

Further to this point, more detail about this research network and group in terms of its long-term plans/objectives would be helpful.

Some of the argumentation in page 7, lines 30-43 - this would benefit from a reference talking about the value of patient experience as evidence. For example: Rand L, Dunn M, Slade I, Upadhyaya S, Sheehan M. Understanding and using patient experiences as evidence in healthcare priority setting. Cost Eff Resour Alloc. 2019 Sep 23;17:20. doi: 10.1186/s12962-019-0188-1. PMID: 31572067; PMCID: PMC6757378. or some of Greenhalgh's publications e.g. "six biases against patients and their carers"

Figure 1- This survey "data" seems to be what the authors are using to support many of their arguments, but this data is not informative. Later in the article, the authors state "numerous projects around patient experience, evaluation and service development were identified by responders to our NMAHP survey" - Does this mean that these were identified as ongoing or previous research that participants have previously been involved in? Or were these identified as research priorities by survey respondents?

Page 7 Lines 54-58- "We are acutely aware of the skills needed by qualitative researchers and the need to demonstrate reflexivity. Our training and practice develop these skills-" this is a key point. But also, what skills are you referring to? I would encourage you to consider exemplars that illustrate this alignment between your practice/educational preparedness as NMAHPS and the skills required of qualitative/engaged/co-design methods researchers. What about skills and practice that prepares NMAHPs to be leaders in co-designed methods (both qualitative and quantitative)

Page 7 Lines 58-60 " essential for approaches such as GT, IPA, interview skills and ethnography.... interviewing could be used in all of these (and other) qualitative methods. I suggest "essential for qualitative interviewing and data analysis"

Page 9 Lines 22-25 and 35-38: these sentences are identical.

Page 9 Lines 46-48 "our survey demonstrates a skilled and motivated workforce who are well-equipped to produce research" - while this may be true, even if this is a commentary article and you cannot present this data, there is no context given that shows how the survey has demonstrated this, only demographics.

If you cannot speak to findings of the survey beyond the occupational demographics of the respondents due to it being a communication article, I suggest you not include it at all.

Perhaps switch the focus to being about the initiative to develop the network of authorship group?

I appreciate the argument that the authors are trying to make related to the need for space and opportunities for NMAHP to do research. I invite the authors to consider if they want to be bold make the comparison of how joint clinical scientist roles and space for these roles writ large is much more status quo in medicine/for MDs, but we need the research conducted by NMAHP who engage with their patients and have an understanding of what research questions and outcomes matter and can work with them in co-production. I understand not wanting to alienate our medical colleagues, but in my eyes, it is more of making the argument for a wider, multidisciplinary seat at the clinical research table; there is a need for our clinical and person-centered angles of vision in research as NMAHPS and there should be space for these roles. This will help disseminate genomics findings and education to our multidisciplinary colleagues as well. While dated, I think this thesis may be relevant

https://open.library.ubc.ca/soa/cIRcle/collections/ubctheses/24/items/1.0383318

I thank the authors for the privilege of knowing their work and for their important initiative and message.

VERSION 1 - AUTHOR RESPONSE

Reviewer: 1
Dr. Stephanie White, University of Technology Sydney
Comments to the Author:
Putting the Patient at the Centre: A Call for Research Involvement of Nurses

Midwives and Allied Health Professionals Working in Genomics

Overall comments

Thank you for the opportunity to review this manuscript that highlights the importance of NMAHPs conducting genomics research to deliver evidence-based, patient-centred care. The authors set out a number of reasons why NMAHPs should be involved in research and the challenges faced in delivering person-centred care. The article would be improved with a more transparent description of their survey, how this is informing the article, and whether they have/need ethics approval to report this data.

We have re-written the paper significantly and removed the survey. The survey was reasonably informal – amongst colleagues. It was used as a way to connect people who felt passionate about GCs and NMAHPS contributing to research. We have now changed the focus on the paper to represent the views of this group, rather than the survey itself.

They also refer to 'discussions' informing the article, and these could be described in better detail (e.g., how many meetings were held, when did they occur, who attended etc). One aspect I felt was missing from the article was the interplay between clinicians, clinician-researchers, and academic researchers, and what opportunities and challenges exist within these structures. The authors mention time as a barrier to conducting research, but there was no explicit discussion about how networking with academic institutions could facilitate research opportunities for those who are primarily working in the clinical setting. Further to this, it's not clear whether the authors are coming to this discussion from an academic or clinical perspective, and this would be helpful to understand in order to contextualise their article. The other missing component was where NMAHP research fits in within the larger body of genomics research. The arguments for increased participation would be strengthened by highlighting what it is specifically that we add to the research agenda. You discuss addressing the psychological and familial aspects of genetics, but it would be good to articulate what our unique lens/training/viewpoint adds to the research already undertaken/underway by other disciplines. You could also highlight the limitations of approaching research through our lens and how we can overcome the challenges (e.g., limited opportunities to lead research groups, or inadequate research training in undergrad/post grad education). Overall, I believe this is an important topic and article, but it requires some refinement before publishing. I hope these comments are helpful and I would be happy to review another version in the future.

We have significantly re-written the article in a way that we hope addresses the points raised above. The section *making space for NMAPS to do research* we hope now clearly outlines the challenges NMAHPs face in contributing to research. We follow this section up with four specific examples where we feel NMAHPs could be valuable as researchers in the genomics space, before finishing with a clear outline of how a research network could support NMAHPs who wish to do more research.

Abstract

Line 49: Is it accurate to say this an unprecedented approach? I can think of many articles that report on group discussions/consensus.

Indeed. This has been taken out.

Figure 1.

The figure or legend could use a little more explanation, perhaps by including the question that was asked alongside the figure. I am assuming that when you say the responders are "published", this means they have conducted a research study and published this in a peer-reviewed journal? A clearer explanation would help. The same goes for 'opted into email group' (not clear what email group you mean or why this is important data to showcase up front) and 'in education or training' (does this refer to entry level qualifications for their profession?).

You also mention that other people came to this discussion who did not respond to the survey, so is there further information about who the group ended up being that contributed to this article?

Note for self: is this data that they have published previously or should they make some reference to whether ethics approval was obtained for this collection and dissemination of data?

We have taken out the survey responses due to reasonable comments by all the reviewers. In the introduction we have outlined who we are and how we came together to write this paper.

Page 7, line 39

You make reference to your survey here and elsewhere, but it's not clear to me whether this article to seeking to report the findings of your survey because you have not provided concrete data, or referenced a previously published study in which your data is described. I think you need to make your intentions explicit somewhere within the article because it's also not clear whether you are meeting ethical requirements around reporting this data. Do you have ethical approval to report data from this survey, or did you receive an exemption from a relevant ethics review committee?

As per previous comments, we have taken out the survey responses as part of the paper and significantly re-written the paper as an opinion/policy piece representing the views of the authors.

Page 7, lines 49 – 60

It's great that the benefits of qual research are highlighted here, but it would be good to complement this with the strengths of quant research and perhaps mixedmethods, particularly as MM are recommended by groups like the Medical Research Council as the preferred methodology for complex interventions and implementation research.

We hope the new section – that outlines four areas that NMAHPS can contribute to research - reflects the broad nature of the contribution NMHAPs can make to research.

Page 8, first two paragraphs

I agree that these are important considerations. In the context of this article, which seeks to advance the involvement of NMAHPs in genomics research, this section would be improved by linking how these challenges relate to the involvement of NMAHPs in research.

This concerns should be addressed in the re-written section: Making space for NMAHPs in research.

Page 10, line 7-9

Can you describe further how NMAHPs are able to reach underrepresented voices that PPIE groups are unable to?

We hope this is now capture in the section *Patient perspective and epistemic justice*

Pag 10, lines 28-30

This section would be strengthened with a more specific description of what your group intends to do. For example, how will you champion the implementation of research findings into practice? Who will you expand your network to and how will this facilitate the outcomes you want to achieve?

We have clarified in the final section *Why is an NMAHP research network important?* What we hope the research network could achieve in a way that I hope addresses the concerns raise above.

Reviewer:2
Dr. Maria Siermann, KU Leuven

Comments to the Author:

General:
- This is an interesting commentary paper that highlights the need for patient-centred research in

genomics and for the involvement of NMAPHs in that research. I think the patient-centred research (and care) is very important, particularly in the mainstreaming of genomics, and therefore commend the efforts of the authors.

- However, the structure of the manuscript could be improved. The aim of the paper is not fully clear. Sometimes it discusses the need for more patient-centred care, sometimes for more patient-centred research, sometimes for more research to be done by NMAPHs, and sometimes for more research to be done by the group of authors. Of course these issues are interrelated, but the specificities and interrelations of these issues are not made very clear. It might be better to choose one area to focus on specifically, and to really argue for that issue specifically (and make the interrelations with the other issues clearer).

We have restructured the paper significantly in light of these comments. We now outline four specific areas where we feel NMAHPs could contribute to research with a discussion about how this could be enabled through the network.

- The aim and content of the survey is not very clear to me. As I have stated in a comment later on, I would opt to either focus more on the results of this paper, or otherwise to leave the survey out and write the manuscript as a commentary paper. This might help streamline the paper and emphasize its goal.

We agree and have left the survey out (other than to explain how it served as a reason for how the writing group came together.)

Abstract & title:

- In the abstract you conclude that "We propose that NMAHPs, with our research expertise, are best placed to shape and deliver a research agenda that explores models of patient centred care in the genomics era. We call for increased funding of NMAHP research roles and funding opportunities to deliver this fundamental work." However, this is different from your conclusion in the paper, where you talk more about ways the move forward, and do not state that NMAHPs are the best placed, but rather that it is crucial that NMAHPs are involved in research and clinical care in genomics.

We have adjusted the abstract to "*NMAHPS are well placed"* and with the restructured article we hope the abstract and conclusion are now more closely aligned.

Introduction:
- Maybe I'm missing something, but I don't see the footnote information for the quote "The Patient will be at the heart of everything the NHS does"1. Who is this quote by?

This quote is taken form the NHS constitution found here

The NHS Constitution for England - GOV.UK

- The introduction is a bit vague and short. I would suggest starting with describing a development or 'problem', to make the aim and need of this paper clear. Or integrate it more with the sections that follow, instead of having separate section/subheadings.

This section has been re-written significantly to address these points

Present and Future Genetics:

- “Evidence about psychosocial impacts of these new types of tests and their consequences for individuals in families who decline testing is limited9.”: What do you mean with ‘these new types of tests’? For some of the newer tests you mention earlier there has been research done about the psychosocial impacts of these tests. Can you discuss some of these impacts (with references)?
- “A recent systematic review of direct-to-consumer genetic tests found consumer satisfaction with these products, healthcare professional scepticism, false positive results, and occasional harm10.”: This sentence is difficult to follow. Can you say a bit more about the type of direct-to-consumer genetic tests you are referring to? And say more about what you mean with scepticism, satisfaction and harm?

Much of this content has been moved to the re-written section *Psychosocial impacts* and the re-worked section hopefully addresses the concerns.

Why is an NMAHP research network important?

- “Numerous projects around patient experience, evaluation and service development were identified by responders to our NMAHP survey”: What do you mean by this? Do you mean projects of the authors of this manuscript? Or are you talking about projects in general? Can you give references?
- “In addition, our group has research expertise in variant interpretation12, clinical outcomes13, 14, guideline development14, 15 and data management16.” and “We expressly assert our readiness to lead on patient centred genomics research, namely, combining quantitative with qualitative approaches. Excellent qualitative research gives patients opportunities to voice authentic experiences. It provides rich datasets and real-world patient insights upon which to base service improvements. We are acutely aware of the skills needed by qualitative researchers and the need to demonstrate reflexivity. Our training and practice develop these skills, which are essential for approaches such as grounded theory, interpretative phenomenological analysis, interview skills and ethnography.” The paper seems to jump from emphasizing the importance of patient-centred research and the role NMAHPs could have in this research in general, to arguing for you as a group specifically being qualified for that research. I think the paper might be stronger by focussing less on your skills as a group, but more on the need for patient-centred research in general and what NMAHPs could add to that research.

Many of the points above revolve around the findings from the survey. We have taken the survey findings out, which hopefully addresses these concerns. We have addressed the need and purpose of a research network in a new section at the end of the paper. We have also more clearly outlined four areas where we feel NMAHP research would be valuable. We hope this provides a clear picture of what we are advocating for in this article.

Challenges to Patient Centred Care - Putting the Patient First:

- “Cascading genetic testing to at-risk relatives allows family members to make active choices towards their healthcare, often before they even develop symptoms. If cascade testing is not facilitated by clinicians, this is a missed opportunity to potentially prevent disease31.”: I think it would be important

to also highlight the fact that not every family member would want to know and be tested. While it can be said that having the option to get tested is important, I think the option of choice is important too. In that sense, getting tested should not only be about disease risk, but also about informed choices. I am also not sure what you mean with the potential to 'prevent disease' in genetics? Are you referring to reproductive selective options?

- Other than that, this section reads really well and seems to be the key part of this manuscript. Much of this content comes under the re-written section *familial nature of genomics* and is one of the key areas we outline for NMAHP research input.

Equity: How do we ensure that testing is available to all?:

- The transition from the previous section to this section could be improved.
- It would be interesting to highlight more that not just access to genetics sercives is not equally available, but that genetics research itself does not ensure equity (think for example about the limited representation of people that are not of European ancestry in biobanks used for genome-wide association studies).

Again, this section has been re-written considerably and now hopefully addresses the concerns raised above.

Making space for NMAHPs to do research:

- It is not clear to me what exactly was asked in the survey and how this "demonstrates a skilled and motivated workforce who are well placed and equipped to produce research that can meet the challenges set out above". I am not doubting that the NMAHPs included are indeed this skilled and motivated workforce, but I am confused about how the survey shows this. Perhaps this survey is not relevant enough for the point you want to make. I think you can argue that putting the patient at the centre of genomics research at the NHS and the need to value the skills of NMAHPs to do just that would be enough of an argument, without a need for the survey.

- Or the other option was to spend more time on the survey results. Now you something refer to these results, but as a reader the results (and aim) of the survey is not very clear.
- Page 10, line 10: Do you mean 'PPIE' rather than 'PPI'?

The paper has been re-written to addresses these concerns. The survey has been taken out. The opening now outlines why NMAHPs can make valuable contributions to research and the final section outlines why the research network we propose could facilitate this.

Conclusion:
- I like that you discuss potential ways to move forward in your discussion.
- However, improving clinical care and patient-centred research seem to be discussed interchangeably. While I realize that research and care goes hand in hand, I think it is important to be clear in what you are discussing and what issue(s) you want to raise.
- While not discussing research in genetics specifically, perhaps the paper 'Ethical preparedness in Genomic medicine: how NHS clinical scientists navigate ethical issues' by Sahan et al. 2024 and our commentary response 'Stimulating professional collective responsibility from the outset in

mainstreaming genomics' could be useful for your manuscript, since similar issues are discussed, though with a different emphasis.

The re-written paper now has a tighter focus on the research areas that we would hope NMAHPS could contribute to, which hopefully is reflected in the conclusion that addresses the concerns raised above.

Reviewer: 3

Dr. Rebecca Puddester, Memorial University of Newfoundland
Comments to the Author:

Thank you to the team for the opportunity to review this commentary article. I think the crux of the arguments of the article are well-worth making.

As a nurse who uses methods of patient engagement in genomics healthcare research, I agree that NMAHP can add tremendous value in the space of engaged/co-design research by virtue of their clinical training and skills that can contribute to person-centred cultures of care. I also believe that there is a need for a culture of effective and two-way transfer of knowledge between research and practice which requires protected time and infrastructure for both activities.

I do think there are a number of things that can be clarified/streamlined to improve this paper and its argumentation. At times, there was repetitive rhetoric that could be enhanced with supportive evidence. Thus, I offer my suggestions in the spirit of collegiality.

Page 4 Line 30- It is not clear to me the details surrounding the survey, why was it sent out, to who sent it out? Was there an option to leave contact information resulting in the formation of this group? I think that more detail surrounding the motivations and circumstances surrounding how this authorship group came together would make it a lot clearer for the reader. Further to this point, more detail about this research network and group in terms of its long-term plans/objectives would be helpful.

We have taken out the survey as part of the manuscript but have clarified how the team came together to write the article. We have also ended the paper with a (hopefully clearer) articulation of what we think the research network can achieve.

Some of the argumentation in page 7, lines 30-43 - this would benefit from a reference talking about the value of patient experience as evidence. For example: Rand L, Dunn M, Slade I, Upadhyaya S, Sheehan M. Understanding and using patient experiences as evidence in healthcare priority setting. Cost Eff Resour Alloc. 2019 Sep 23;17:20. doi: 10.1186/s12962-019-0188-1. PMID: 31572067; PMCID: PMC6757378. or some of Greenhalgh's publications e.g. "six biases against patients and their carers"

We have included this reference in the new section titles *Patient Perspectives and epistemic justice*

Figure 1- This survey "data" seems to be what the authors are using to support many of their

arguments, but this data is not informative. Later in the article, the authors state "numerous projects around patient experience, evaluation and service development were identified by responders to our NMAHP survey" - Does this mean that these were identified as ongoing or previous research that participants have previously been involved in? Or were these identified as research priorities by survey respondents?

We have taken out the survey responses from the article.

Page 7 Lines 54-58- "We are acutely aware of the skills needed by qualitative researchers and the need to demonstrate reflexivity. Our training and practice develop these skills-" this is a key point. But also, what skills are you referring to? I would encourage you to consider exemplars that illustrate this alignment between your practice/educational preparedness as NMAHPS and the skills required of qualitative/engaged/co-design methods researchers. What about skills and practice that prepares NMAHPs to be leaders in co-designed methods (both qualitative and quantitative)

The paper has been significantly re-written. We now outline four areas where NMAHPs could contribute to research and hopefully these new areas capture and present more clearly how we feel NMAHPS skills and practice and usefully contribute to research.

Page 7 Lines 58-60 " essential for approaches such as GT, IPA, interview skills and ethnography.... interviewing could be used in all of these (and other) qualitative methods. I suggest "essential for qualitative interviewing and data analysis"

Page 9 Lines 22-25 and 35-38: these sentences are identical.

Both these sections have been significantly re-written

Page 9 Lines 46-48 "our survey demonstrates a skilled and motivated workforce who are well-equipped to produce research" - while this may be true, even if this is a commentary article and you cannot present this data, there is no context given that shows how the survey has demonstrated this, only demographics.

If you cannot speak to findings of the survey beyond the occupational demographics of the respondents due to it being a communication article, I suggest you not include it at all. Perhaps switch the focus to being about the initiative to develop the network of authorship group?

We have changed the focus of the paper – taking out the survey data - and it now reads as an opinion piece representing the views of the authors.

I appreciate the argument that the authors are trying to make related to the need for space and opportunities for NMAHP to do research. I invite the authors to consider if they want to be bold make the comparison of how joint clinical scientist roles and space for these roles writ large is much more status quo in medicine/for MDs, but we need the research conducted by NMAHP who engage with their patients and have an understanding of what research questions and outcomes matter and can work with them in co-production. I understand not wanting to alienate our medical colleagues, but in my eyes, it is more of making the argument for a wider, multidisciplinary seat at the clinical research table; there is a need for our clinical and person-centered angles of vision in research as NMAHPS and there should be space for these roles. This will help disseminate genomics findings and education to our multidisciplinary colleagues as well. While dated, I think this thesis may be relevant

https://open.library.ubc.ca/soa/cIRcle/collections/ubctheses/24/items/1.0383318 [open.library.ubc.ca]

These are valuable points. The re-written section *Making space for NMAHPs to do research* and (again re-written) section at the end of the paper outlining how the research network can contribute to this aim does – we hope – address the issues raised in the paragraph above

I thank the authors for the privilege of knowing their work and for their important initiative and message.

VERSION 2 - REVIEW

**Reviewer**	**2**
**Name**	**Siermann, Maria**
**Affiliation**	**KU Leuven**
**Date**	**14-Apr-2025**
**COI**	

Well done on the changes made! The manuscript is now a lot clearer and has a coherent and important message. I just have some minor comments.

Comments:

- I would change "The paper aims to set out in clear terms the value of NMAHPs to research that support patients centred implementation of genomics in the NHS" to "The paper aims to set out in clear terms the value of NMAHPs to research that supports patient-centred implementation of genomics in the NHS".

- "This is often referred to as 'mainstreaming' [6] and test criteria, including the specialists authorised to order the test, are set out in the National Test Directory [7].": I would split up this sentence into two sentences.

- The use of capital letters sometimes seems a bit random, such as in this section: "The available avenues for which individuals can receive genetic information have rapidly increased [9], including genotype-first approaches, such as additional findings from the 100,000 genomes project, Newborn Screening for Genetic conditions (the Generation Study) and the abundant commercial availability of 'direct-to-consumer' genetic tests. The variety and purpose of genetic testing are also increasing beyond diagnosis. For example, Pharmacogenomics is driving personalised treatment and improving drug safety and efficacy [10]." I would suggest to be consistent with when words are and are not capitalized.

- What exactly do you mean by "professions outside of Medicine"? Are you referring to people without a degree in medicine?

- A full stop is missing after reference 15 in the text.

- The ‘Who we are and how we came together’ section is now explained multiple times, which I don’t think is necessary. Perhaps the ‘Introduction’ and ‘Who we are and how we came together’ can be removed from the start of the paper, or the explanation of how this group came together can be removed later on.

- “to consent patients” sounds a bit strange to me.

- “the challenge of accurate and phenotyping”: either a word is missing or ‘and’ can be deleted.

- You mention that there are certain types of genetic/genomic tests that might require less counseling. Are there also certain tests that require more counseling perhaps (where NMAHPs would be needed)? I would also mention the increasing use of genomics in population screening in that case (where people don’t come for a specific diagnosis but might receive unexpected and/or large amounts of genomic information).

- I think the complexities of family communication in genomics, including ethical and policy aspects, deserve some more attention in the section on ‘Familial nature of genomics’.

- The lack of equality in representation in genomics research and the lack of equality in access to and experiences of genomic tests can be interrelated, but are also separate issues. It would help for the clarity of the manuscript to be clearer about this in the equity section (now for example “some groups have historically, and still remain, significantly underrepresented” reads strangely in this sentence).

- The role of NMAHPs in improving (research on) equity in genomics could be described more clearly (and extensively).

- “People from the deaf community, the autistic community and those writing from a disability rights perspective, for example, have all argued that genetic testing can serve to reinforce discriminatory attitudes and prejudicial medical models of disability.” This could use references.

- “tackle issues of epistemic injective” should be “tackle issues of epistemic injustice”.

- Would the tackling of epistemic injustice, next to NMAHPs, also include/involve those communities you mentioned?

**Reviewer**	**3**
**Name**	**Puddester, Rebecca**
**Affiliation**	**Memorial University of Newfoundland**
**Date**	**21-Apr-2025**
**COI**	

Thank you to the authors for the opportunity to review this manuscript again, now revised as a commentary paper. This pivot has been effective—the argumentation is clearer, and the focus is more streamlined and compelling. The points raised are important and timely, and I commend the authors for their thoughtful work. I recommend accepting the paper with minor revisions, with one key suggestion related to the philosophical framing and organization of the “four areas.”

Main Comment – Restructuring with the Philosophical Framework:

The current fourth area—patient perspectives and epistemic justice (which is entirely appropriate)—but in my view, transcends the other three (psychological impacts, the familial nature of genomics, and equity).

Suggested Structural Revision:

I encourage the authors to consider presenting patient perspectives and epistemic injustice first (shortly after the content in the 'Making Space" section...") as a guiding framework that grounds the remainder of the commentary. This repositioning would provide a compelling “raison d’être” for why the contributions of NMAHPs are particularly valuable in genomics research and integration. Framing epistemic injustice in this way would help unify the subsequent discussion of psychosocial impact, family dynamics, and equity as specific domains where epistemic injustice may arise—if patient perspectives are not considered- and where NMAHPs are uniquely equipped to intervene/contribute.

This also invites a reconsideration of the subsection titled “Implementing genomics: barriers & challenges”. As currently framed (p.6, lines 3–25), this section feels slightly misaligned with the central argument of the commentary. A revised framing, such as “What benefits are NMAHPs poised to bring to person-centred genomics research?” or “What gaps are NMAHPs positioned to fill?” might better reflect the thrust of the paper.

Related to this, within that section, the first paragraph (lines 3–12) could be streamlined to something like:

“While the promises of genomics are vast, they also introduce sever several system-wide challenges and unprecedented complexities accompany the implementation of genomics (e.g., sample logistics, interpretation of findings, consent processes), amid these challenges and complexities- it is essential to consider how these complexities affect patients and families, and how their preferences and lived experiences should inform research/implementation"

This could then serve as a natural segue into the discussion of patient perspectives and the risks of epistemic injustice, especially when lay or lived-experience voices are excluded from genomic research and policy.

Additionally, (if the authors uptake my suggestions), I see potential for some of the content from p.8, lines 45–54 (currently within the epistemic injustice discussion) to be incorporated into the equity section instead—since these ideas intersect closely with access and inclusion.

Summary of Structural Suggestion:

Reframe epistemic injustice and patient perspectives as a foundational framework, introduced early in the commentary

Follow with the three content areas/or opportunities where lived experience data should be considered

Psychosocial impact

Familial nature of genomics

Equity

Re-title the "barriers & challenges" section to reflect the unique role or potential contributions of NMAHPs in person-centered perspectives

I believe this reordering and slight re-framing is a very minor change and will make your argument even more compelling and cohesive. Thank you again for the opportunity to review this important and timely piece and I look forward to seeing it in print.

All the best,

VERSION 2 - AUTHOR RESPONSE

Reviewer: 2
Dr. Maria Siermann, KU Leuven
Comments to the Author:
Well done on the changes made! The manuscript is now a lot clearer and has a coherent and important message. I just have some minor comments.

We thank the reviewer for your thoughtful and helpful comments in clarifying our paper. We give our responses to each comment below.

Comments:
I would change "The paper aims to set out in clear terms the value of NMAHPs to research that support patients centred implementation of genomics in the NHS" to "The paper aims to set out in clear terms the value of NMAHPs to research that supports patient-centred implementation of genomics in the NHS".

We agree this is clearer, and have changed accordingly lines 20- 21

- "This is often referred to as 'mainstreaming' [6] and test criteria, including the specialists authorised to order the test, are set out in the National Test Directory [7].": I would split up this sentence into two sentences.

Thank you. We have split up this sentence, as suggested, lines 68-71 and agree that it now reads clearer.

- The use of capital letters sometimes seems a bit random, such as in this section: "The available

avenues for which individuals can receive genetic information have rapidly increased [9], including genotype-first approaches, such as additional findings from the 100,000 genomes project, Newborn Screening for Genetic conditions (the Generation Study) and the abundant commercial availability of 'direct-to-consumer' genetic tests. The variety and purpose of genetic testing are also increasing beyond diagnosis. For example, Pharmacogenomics is driving personalised treatment and improving drug safety and efficacy [10]." I would suggest to be consistent with when words are and are not capitalized.

Thank you. We have amended this section to provide more consistency with capitalisation lines 77-83

- What exactly do you mean by "professions outside of Medicine"? Are you referring to people without a degree in medicine?

We have clarified what we mean here – professionals other than doctors lines 87-89

- A full stop is missing after reference 15 in the text.

Thank you. We have added this in line 105

- The 'Who we are and how we came together' section is now explained multiple times, which I don't think is necessary. Perhaps the 'Introduction' and 'Who we are and how we came together' can be removed from the start of the paper, or the explanation of how this group came together can be removed later on.

Thank you. Indeed, this was repetition. This is now outlined only once at the start of the paper line 41.

- "to consent patients" sounds a bit strange to me.

We have amended this section – now found under the paragraph titled *implementing genomics* to improve the clarity. We have amended the phrase 'to take consent' line 131-132.

- "the challenge of accurate and phenotyping": either a word is missing or 'and' can be deleted.

Thank you we have amended to: 'Other barriers identified include the challenge of accurate phenotyping' line 133-135

- You mention that there are certain types of genetic/genomic tests that might require less counseling. Are there also certain tests that require more counseling perhaps (where NMAHPs would be needed)? I would also mention the increasing use of genomics in population screening in that case (where people don't come for a specific diagnosis but might receive unexpected and/or large amounts of genomic information).

We have addressed your helpful point to highlight where certain tests may require more counselling by NMAHPs. We have given the example of prenatal testing (line 206-208). We agree that population screening resulting in unexpected findings is a useful addition and have included this. It

can be found in the penultimate paragraph on the section *Psychosocial impacts line 214-217.* Thank you.

- I think the complexities of family communication in genomics, including ethical and policy aspects, deserve some more attention in the section on 'Familial nature of genomics'.

We agree that this is an area where there are significant ethical and policy aspects that could be discussed in further detail. A challenge with each of the four areas of interest we outline is that they could warrant a paper in their own right. As such, we have tried to focus on how they illustrate the potential value of improving NMAHP's contribution to research.

Thank you. We have added a paragraph line 235-239 to better reflect complex ethical and policy issues in this area and to place our discussion more clearly in the context of the point we are trying to make about NMAHP participation in research.

- The lack of equality in representation in genomics research and the lack of equality in access to and experiences of genomic tests can be interrelated, but are also separate issues. It would help for the clarity of the manuscript to be clearer about this in the equity section (now for example "some groups have historically, and still remain, significantly underrepresented" reads strangely in this sentence).

Thank you for making this distinction. We have clarified this section to draw a distinction between equality as it relates to access to clinical services and as it relates to representation in research line 263-264.

- The role of NMAHPs in improving (research on) equity in genomics could be described more clearly (and extensively).

We have added a paragraph at the end of this section to more clearly articulate why we feel NMAHPs are an important workforce to tackle the area through participation in research line 279-286

- "People from the deaf community, the autistic community and those writing from a disability rights perspective, for example, have all argued that genetic testing can serve to reinforce discriminatory attitudes and prejudicial medical models of disability." This could use references.

We agree and have included appropriate references line 167

- "tackle issues of epistemic injective" should be "tackle issues of epistemic injustice".

Thank you, we have amended this line 184-185

- Would the tackling of epistemic injustice, next to NMAHPs, also include/involve those communities you mentioned?

We agree with this point and have made it more explicit that we see NMAHPs as an ideal workforce for this purpose line 179-181

Reviewer: 3
Dr. Rebecca Puddester, Memorial University of Newfoundland
Comments to the Author:

Thank you to the authors for the opportunity to review this manuscript again, now revised as a commentary paper. This pivot has been effective—the argumentation is clearer, and the focus is more streamlined and compelling. The points raised are important and timely, and I commend the authors for their thoughtful work. I recommend accepting the paper with minor revisions, with one key suggestion related to the philosophical framing and organization of the “four areas.”

Thank you for your encouraging feedback, comments and suggestions helping us to clarify and refine our paper. We very much appreciate your time and effort. As your suggestions are mainly around restructuring, it was difficult to respond line by line. Please find our responses below.
Main Comment – Restructuring with the Philosophical Framework:

The current fourth area—patient perspectives and epistemic justice (which is entirely appropriate)—but in my view, transcends the other three (psychological impacts, the familial nature of genomics, and equity).
Suggested Structural Revision:

I encourage the authors to consider presenting patient perspectives and epistemic injustice first (shortly after the content in the 'Making Space" section...") as a guiding framework, that grounds the remainder of the commentary. This repositioning would provide a compelling “raison d’être” for why the contributions of NMAHPs are particularly valuable in genomics research and integration. Framing epistemic injustice in this way would help unify the subsequent discussion of psychosocial impact, family dynamics, and equity as specific domains where epistemic injustice may arise—if patient perspectives are not considered- and where NMAHPs are uniquely equipped to intervene/contribute.

Thank you for the comments above. We agree on the importance of epistemic injustice. We have moved this section so it now has more prominence – coming first in the discussion of the ‘four themes’ Line 146 onwards. In many ways, this theme permeates much of the rest of the article. Many of the research areas outlined below are ones where epistemic injustice could arise. In each of these areas, addressing epistemic justice could serve as a central theme and it is our opinion that NMAHPs are well placed as a group to addresses this issue. However, in this article the central idea is to argue for an increased participation of NMAHPs in research. NMAHPs contribution to research is likely to be broad – representing the diversity of the workforce. As such we present four areas as standalone; relevant to but distinct from the theme of epistemic injustice. This allows us to make a broad – and hence the strongest case possible- to demonstrate the value of NMAHPs in research.

This also invites a reconsideration of the subsection titled “Implementing genomics: barriers & challenges”. As currently framed (p.6, lines 3–25), this section feels slightly misaligned with the central argument of the commentary. A revised framing, such as “What benefits are NMAHPs poised to bring to person-centred genomics research?” or “What gaps are NMAHPs positioned to fill?” might better reflect the thrust of the paper.

With regard to the restructure, we have taken the section *Implementing genomics: Barriers and enablers* and changed this to *Implementing genomics line 126*. We have then re-worked this to contextualise more clearly how we see the four areas we outline and how these serve the broader argument we are making.

We believe this restructure adds clarity, giving more prominence to epistemic injustice and we hope this satisfactorily addresses the reviewers concerns.

Related to this, within that section, the first paragraph (lines 3–12) could be streamlined to something like:
“While the promises of genomics are vast, they also introduce sever several system-wide challenges and unprecedented complexities accompany the implementation of genomics (e.g., sample logistics, interpretation of findings, consent processes), amid these challenges and complexities- it is essential to consider how these complexities affect patients and families, and how their preferences and lived experiences should inform research/implementation"

This could then serve as a natural segue into the discussion of patient perspectives and the risks of epistemic injustice, especially when lay or lived-experience voices are excluded from genomic research and policy.

Additionally, (if the authors uptake my suggestions), I see potential for some of the content from p.8, lines 45–54 (currently within the epistemic injustice discussion) to be incorporated into the equity section instead—since these ideas intersect closely with access and inclusion.

Thank you for the thoughtful comments. We believe that much of our response to these points is covered in the text above. We agree that epistemic injustice is an important topic. We agree, that this paper could be restructured placing this as a central theme, exploring the role of NMAHPs to address this.

It is our view that this would not only be a paper in its own right but also a different one from what we set out to argue. Our argument is broader – that NMAHPs are an underused resource in the research genomics space. As such the four areas we outline are in service of this argument. To restructure as the reviewer suggests would switch this –it would be a paper in which we argue for increased NMAHPs in service of tackling epistemic injustice. We agree that this would make a great paper! However, the idea is different from the one we set out to highlight in this article.

Summary of Structural Suggestion:

Reframe epistemic injustice and patient perspectives as a foundational framework, introduced early in the commentary

Follow with the three content areas/or opportunities where lived experience data should be considered

Psychosocial impact

Familial nature of genomics

Equity

Re-title the “barriers & challenges” section to reflect the unique role or potential contributions of NMAHPs in person-centered perspectives

The paragraph ‘implementing genomics’ is a re-worked version of the paragraph ‘implementing genomics; barriers and enablers.’ This now provides a clearer introduction for the four areas we highlight as demonstrating the value of NMAHPs to research. We have moved the section ‘patient perspectives and epistemic justice’ to earlier in the paper for higher prominence. Thank you for your suggestions.

VERSION 3 - REVIEW

**Reviewer**	**2**
**Name**	**Siermann, Maria**
**Affiliation**	**KU Leuven**
**Date**	**02-Jul-2025**
**COI**	

Thank you for making the minor changes and well done on this interesting paper! I have no further comments.

**Reviewer**	**3**
**Name**	**Puddester, Rebecca**
**Affiliation**	**Memorial University of Newfoundland**
**Date**	**03-Jul-2025**
**COI**	

Thank you for the opportunity to review this paper and the authors' responses.

As I noted in my initial review, its advocacy message for NMAHP involvement in genomics research is a needed one. I also think that reporting of this collaborative network belongs in the published literature to inspire similar activity globally. The description of this group reads even clearer in this version.

The point by the authors that their message was broader the EJ is well taken.

I apologize to the authors that my comments were not as clear as they could have been during R2. My main point (which is sufficiently addressed now in how these subsections are structured) was that it is in capturing/privileging 'patient perspectives' (whether they are collected through either PPE engaged approaches, or any qualitative or quantitative

PROMS/PREMS that capture lived experience data) how we arrive research findings surrounding psychosocial impacts and the familial nature of genomics.

All of which support the main argument that that NMAHP are well suited in genomics research to elucidate these perspectives.

Thanks to the authors and well done with this contribution.
